# Adaptive Joint Carrier and DOA Estimations of FHSS Signals Based on Knowledge-Enhanced Compressed Measurements and Deep Learning

**DOI:** 10.3390/e26070544

**Published:** 2024-06-26

**Authors:** Yinghai Jiang, Feng Liu

**Affiliations:** 1 College of Electronic Information and Optical Engineering, Nankai University, Tianjin 300350, China; 2 Tianjin Key Laboratory of Optoelectronic Sensor and Sensing Network Technology, Nankai University, Tianjin 300350, China

**Keywords:** knowledge-enhanced compressed measurements, FHSS, carrier estimation, direction-of-arrival estimation, deep learning

## Abstract

As one of the most widely used spread spectrum techniques, the frequency-hopping spread spectrum (FHSS) has been widely adopted in both civilian and military secure communications. In this technique, the carrier frequency of the signal hops pseudo-randomly over a large range, compared to the baseband. To capture an FHSS signal, conventional non-cooperative receivers without knowledge of the carrier have to operate at a high sampling rate covering the entire FHSS hopping range, according to the Nyquist sampling theorem. In this paper, we propose an adaptive compressed method for joint carrier and direction of arrival (DOA) estimations of FHSS signals, enabling subsequent non-cooperative processing. The compressed measurement kernels (i.e., non-zero entries in the sensing matrix) have been adaptively designed based on the posterior knowledge of the signal and task-specific information optimization. Moreover, a deep neural network has been designed to ensure the efficiency of the measurement kernel design process. Finally, the signal carrier and DOA are estimated based on the measurement data. Through simulations, the performance of the adaptively designed measurement kernels is proved to be improved over the random measurement kernels. In addition, the proposed method is shown to outperform the compressed methods in the literature.

## 1. Introduction

As effective methods to enhance the resistance capacity of signals against interference and interception, spread spectrum (SS) technologies have been adopted in various fields, including military communications, civilian secure communications, and wireless networks. In these technologies, the spectra of the baseband signals are expanded to a much broader range. Consequently, without significant power covering the entire spread bandwidth, common interference signals can only affect a small portion of the spectra of SS signals.

Frequency-hopping spread spectrum (FHSS) signals are a particular category of SS signals. They have robust anti-interception capacities [1,2] employing pseudo-random carrier frequency-hopping sequences. For non-cooperative receivers, in order to catch the FHSS signals and localize their transmitters, parameter estimations, especially carrier and direction of arrival (DOA) estimations, have to be performed first. Therefore, extensive research has been conducted in this area in recent years.

From the aspect of signal analysis, the transform-based methods, including short-time Fourier transform (STFT)-based [3,4,5,6], wavelet transform-based [7,8,9,10], and autocorrelation analysis-based [11] methods, are the most straightforward approaches for signal parameter estimations. Even in recent years, transform-based methods have continued to be extensively developed. For example, in 2019, Wan et al. [12] proposed a blind parameter estimation algorithm of FHSS signals based on space-time frequency analysis and matrix joint diagonalization. More recently, Jiang et al. [13] designed a kernel function of the time-frequency transform to obtain the time-frequency distribution of the FHSS signal, as well as a model to extract the frequency-hopping ridge, which was used to estimate the signal parameters. In addition to the transform-based methods, energy- or statistical property-based methods, such as the channelized energy thresholding-based [14,15,16], sub-band occupation likelihood analysis-based [17,18] and spectrum analyzer-based [19,20,21] methods, are also implemented in FHSS signal parameter estimations, especially in carrier estimations. In recent years, prosperous development of methods in this category has also been presented. For example, in 2015, Zhang et al. [22] designed a method based on multilevel channelized processing to detect and estimate the carrier of FHSS signals in a complicated electromagnetic environment. In 2023, Li et al. [23] proposed a method combining the maximum likelihood theory with orthogonal matching pursuit to estimate the carriers of multiple FHSS signals. Although the above methods can achieve satisfactory performance, they require high sampling rates determined by the bandwidths of interest, according to the Nyquist sampling theory. With the increasingly widening bandwidths for FHSS techniques, these methods would face challenges on hardware, in terms of sampling rates and computational complexities.

The rendering of the compressed sensing (CS) theory [24,25] provides potential solutions to the problems of sampling rate and computational complexity. According to the CS theory, a signal can be perfectly reconstructed with overwhelming probability from significantly fewer samples than those suggested by the Nyquist sampling theory, if it can be sparsely represented through a transform or a dictionary. As the FHSS signals show significant sparsity in the time-frequency domain, more and more research efforts have been devoted to the exploitation of the CS theory for the processing of these signals [26,27].

To conduct the CS operations, random demodulation [28], multi-coset [29,30], and modulated wideband converter (MWC) [31,32] have been implemented to reduce the sampling rates in recent years. Moreover, with the development of antenna array technology, CS-based methods have also been developed for DOA estimations of FHSS signals. In 2017, Ioushua et al. [33] introduced an MWC-based system composed of an L-shaped antenna array, which can jointly estimate the carrier frequency and DOA of FHSS signals compressively. Additionally, Lei et al. [34] proposed an algorithm for joint spectrum sensing and DOA estimation, based on a simplified MWC structure. In 2021, Zhang et al. [35] proposed an algorithm based on the CANDECOMP/PARAFAC decomposition and the multiple signal classification (MUSIC) in an MWC-based simplified array receiver. However, as the performances of those works were particularly impacted in low signal-to-noise ratio (SNR) scenarios, challenges still remain. Furthermore, as real-time FHSS processing is usually needed, efficient algorithms for quick signal analysis are still required to be developed with certain hardware conditions.

Nowadays, with the outstanding computational power of graphics processing units (GPUs), deep learning technology has also been widely adopted in signal processing areas, including signal feature extractions [36], classifications [37], and parameter estimations [38]. In these implementations, the trained deep neural networks (DNNs) showed high efficiency and outstanding signal analysis capabilities [39,40]. Additionally, efforts have also been devoted to the study of the improvement of DNN robustness [41,42]. However, as an intrinsic problem of trained DNNs, adaptability during their implementations has remained limited.

In this paper, we propose an adaptive method to jointly estimate the FHSS carrier and DOA using compressed sampling rates. In this method, a compressed measurement framework with an antenna array is rendered. The compressed measurement kernels (i.e., non-zero coefficients in the measurement matrix) are optimized based on the task-specific information (TSI) optimization and the continuously updated posterior knowledge of the signal, while a DNN is trained to ensure the efficiency of the measurement kernel design. The carrier and DOA are estimated from the measurement data. The main contributions of this work are as follows:

1. Compared to existing works, the proposed method can directly obtain the frequency and DOA of the FHSS signal from compressed samples with improved performance, without the need for signal reconstruction or high-rate transform/inverse transforms.

2. The proposed method integrates TSI optimization in the design of subsequent measurement kernels, utilizing the obtained measurement data from the antenna array. Therefore, the accuracy of the FHSS carrier and DOA estimations is enhanced, especially for low-SNR scenarios.

3. The proposed method replaces the inefficient recursive design process of measurement kernels with the implementation of a trained neural network. Therefore, the repeated complex online measurement kernel optimization in the adaptive measurements is replaced with one-time offline DNN training and repeated online DNN implementation, which greatly accelerates the measurement process and improves the system’s applicability.

The remainder of this paper is organized as follows: In Section 2, the problem formulation is presented, introducing a framework to implement the proposed method, as well as the signal and CS models. In Section 3, the adaptive measurement kernel design method, including theoretical analysis from the aspect of TSI optimization and the implementation of deep learning, is detailed. Then, in Section 4, a method to estimate the carrier and DOA of the FHSS signal from compressed measurements is described. The simulation results are shown in Section 5. Finally, our conclusions are drawn in Section 6.

## 2. Problem Formulation

**Figure 1:**
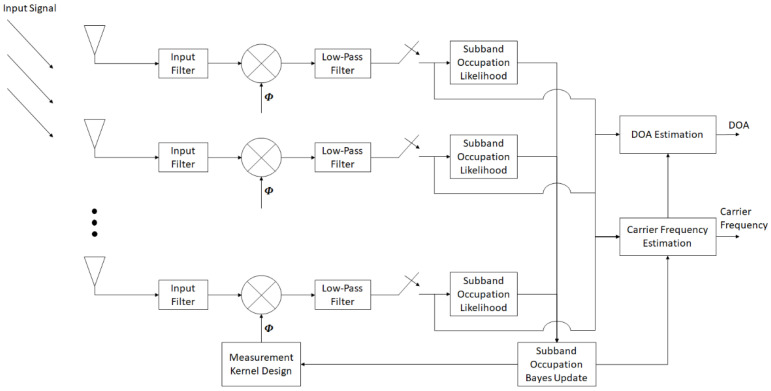
The proposed framework for carrier and DOA estimations of the FHSS signal based on adaptive compressed measurements.

In this paper, we focus on the framework and algorithm development for non-cooperative carrier and DOA estimations of the FHSS signal, with the combination of knowledge-enhanced compressed measurements and deep learning. The framework to conduct the proposed method is shown in Figure 1:

As shown in Figure 1, an antenna array with *K* elements is implemented, and the signals from the antenna elements are first preprocessed through the input filters to eliminate the components beyond the band of interest. The filtering results are then multiplied with the measurement kernels and subsequently passed through low-pass filters, which play the roles of integrators. To obtain the compressed measurements, the results from the low-pass filters are sampled at a compressed rate, which is much lower than that indicated by the Nyquist sampling theory regarding the frequency-hopping range.

To implement this framework, the entire FHSS hopping range is uniformly divided into $N_{b}$ sub-bands. $N_{b}$ can be decided according to the FHSS hopping range. With sufficient computational resources, larger FHSS hopping ranges require more sub-bands, in order to achieve satisfactory algorithm performance. Then, a likelihood analysis of frequency sub-band occupation is performed using the compressed measurements from each antenna element. The likelihood analysis results are employed to update the posterior probabilities of sub-band occupation for the FHSS signal. These posterior probabilities are then used to design the measurement kernel for compressed measurement in the next step.

In addition to the measurement kernel design, the posterior sub-band occupation probabilities are also utilized in conjunction with the measurement data for carrier estimation of the FHSS signal. Finally, the estimated carrier and the measurement data are used to estimate the DOA of the FHSS signal.

In this paper, the elements of the antenna array are coherent and, thus, the same compressed measurement kernels are used for all of the antenna elements in each measurement step. We assume that the signal of interest is a far-field FHSS signal, resulting in planar-wave signals. As the carrier frequency hops within a wide band, the signal can be regarded as narrow-band within each frequency-hopping cycle. Therefore, the envelope of the baseband signal can also be considered to be slowly altered. Let us represent the non-compressed baseband signal according to the FHSS hopping range with a column vector s and assume that the communication channel is an additive Gaussian white noise channel. Then, the signal received by the $k^{\mathrm{th}}$ antenna element (k=1,2,…,K) can be expressed as

(1) $$x_{k}=s_{k}+n_{k}=sa_{k}\left(\Omega \right)+n_{k},$$

where $a_{k}\left(\Omega \right)=\exp\left[-j2\pi f_{c}\Delta t_{k}\left(\Omega \right)\right]$ is the $k^{\mathrm{th}}$ element in the steering vector of the antenna array, $f_{c}$ is the carrier frequency of the signal, $\Delta t_{k}$ is the time delay of the signal received on the $k^{th}$ antenna element, relative to the reference point, and $n_{k}$ represents the additive noise on the $k^{\mathrm{th}}$ antenna element with the variance of $\sigma_{n}^{2}$.

In the path after each antenna element in Figure 1, the compressed sensing can be expressed as

(2) $$y_{k}=\Phi x_{k}=\Phi \left(s_{k}+n_{k}\right),$$

where Φ is the M×N sensing matrix, with M<N. By implementing the framework in Figure 1, Φ becomes a block diagonal with each block as a 1×R row vector (i.e., a measurement kernel), where $R=\frac{N}{M}$ represents the compression ratio.

In order to demonstrate the proposed method, the measurement kernel design approach and the carrier/DOA estimations approach are detailed in the following two sections, respectively.

## 3. Adaptive Measurement Kernel Design with the Combination of TSI Optimization and Deep Learning

### 3.1. Measurement Kernel Design with TSI Optimization

In the framework proposed in Figure 1, the signal is measured compressively at regular intervals. In this scenario, the first measurement kernel is taken as a normalized random vector, and the subsequent measurement kernels are adaptively designed based on the measurement data that have already been obtained. In particular, to design the measurement kernel for the $m^{\mathrm{th}}$ (1 < *m* ≤ *M*) measurement, the posterior probability density function (PDF) of the signal, given the $1^{\mathrm{st}}$ through the ${\left(m-1\right)}^{\mathrm{th}}$ measurements, is modeled as

(3) $$p_{r}\left(s^{m}|P^{m-1}\right)=\sum_{l=1}^{N_{b}}P_{l}^{m-1}f_{s,l}\left(s^{m}\right),$$

where $f_{s,l}\left(s^{m}\right)$ is a Gaussian white component that covers the $l^{\mathrm{th}}$ ($l=1,2,\ldots ,N_{b}$) sub-band, with zero mean and the covariance matrix denoted by $\Xi_{s,l}$. $P^{m-1}={\left[P_{1}^{m-1},P_{2}^{m-1},\ldots ,P_{N_{b}}^{m-1}\right]}^{T}$, with $P_{l}^{m-1}$ representing the posterior probability that the $l^{\mathrm{th}}$ sub-band is occupied, given the $1^{\mathrm{st}}$ through the ${\left(m-1\right)}^{\mathrm{th}}$ measurements, $P_{l}^{0}=\frac{1}{N_{b}}$.

In order to reduce the computational complexity at this point, by ignoring the correlation among the measurements from different antenna elements and the measurements at different steps, $P_{l}^{m-1}$ (1 < *m* ≤ *M*) is obtained by a Bayes update with $P_{l}^{m-2}$ and the ${\left(m-1\right)}^{\mathrm{th}}$ measurement from each antenna element, using

(4) $$P_{l}^{m-1}=\frac{P_{l}^{m-2}\prod_{k=1}^{K}f_{y,l,m-1}\left(y_{k}^{m-1}\right)}{\sum_{l=1}^{N_{b}}P_{l}^{m-2}\prod_{k=1}^{K}f_{y,k,m-1}\left(y_{k}^{m-1}\right)},$$

where $y_{k}^{m-1}=\Phi^{m-1}x_{k}^{m-1}$ represents the ${\left(m-1\right)}^{\mathrm{th}}$ compressed measurement result at the $k^{\mathrm{th}}$ antenna element, with $\Phi^{m-1}$ and $x_{k}^{m-1}$ standing for the measurement kernel and the input signal, respectively; $x_{k}^{m-1}=s^{m-1}a_{k}\left(\Omega \right)+n_{k}^{m-1}$, where $s^{m-1}$ and $n_{k}^{m-1}$ represent the FHSS signal of interest and the additive noise at the ${\left(m-1\right)}^{\mathrm{th}}$ measurement of the $k^{\mathrm{th}}$ antenna element, respectively. Let us denote $I_{R}$ as an R×R identity matrix. Then, $f_{y,k,m-1}\left(y_{k}^{m-1}\right)$ is a zero-mean Gaussian component, with the variance of $\sigma_{y,k,m-1}^{2}=\Phi^{m-1}\left(\Xi_{s,l}+\sigma_{n}^{2}I_{R}\right){\Phi^{m-1}}^{H}$, where ${\left(\cdot \right)}^{H}$ stands for the Hermitian operation of a matrix.

Let us denote $y^{m}={\left[y_{1}^{m},y_{2}^{m},\ldots ,y_{K}^{m}\right]}^{T}$ as the collection of the $m^{\mathrm{th}}$ measurements from the antenna elements, where ${\left(\cdot \right)}^{T}$ stands for the transform operation of a matrix. Then, we define conditional mutual information $I(s^{m};y^{m}|P^{m-1},\Phi^{m})$ as the TSI. In this scenario, the design of the $m^{\mathrm{th}}$ measurement kernel is performed by solving the following optimization problem:

(5) $${\hat{\Phi }}^{m}={\mathrm{argmax}}_{\Phi^{m}}I\left(s^{m};y^{m}|P^{m-1},\Phi^{m}\right),s.t.{∥\Phi^{m}∥}_{l_{2}}=1,$$

where ${∥\cdot ∥}_{l_{2}}$ denotes the l−2 norm operation.

According to information theory, it is known that $I(s^{m};y^{m}|P^{m-1},\Phi^{m})$ can be expressed by the difference between two conditional entropies:

(6) $$I\left(s^{m};y^{m}|P^{m-1},\Phi^{m}\right)=h\left(y^{m}|P^{m-1},\Phi^{m}\right)-h\left(y^{m}|s^{m},P^{m-1},\Phi^{m}\right).$$

In Equation (6), as ${∥\Phi^{m}∥}_{l_{2}}=1$, the entries of $y^{m}$ are independently Gaussian-distributed with the variances of $\sigma_{n}^{2}$, given the signal $s^{m}$. Therefore, $h(y^{m}|s^{m},P^{m-1},\Phi^{m})$ is a constant. Additionally, by ignoring the correlations among the received signals from different antenna elements, it can be ascertained that $h\left(y^{m}|P^{m-1},\Phi^{m}\right)=\sum_{k=1}^{K}h\left(y_{k}^{m}|P^{m-1},\Phi^{m}\right)=Kh\left(y_{k}^{m}|P^{m-1},\Phi^{m}\right)$. Therefore, the optimization problem of Equation (5) is equivalent to

(7) $${\hat{\Phi }}^{m}={\mathrm{argmax}}_{\Phi^{m}}h\left(y_{k}^{m}|P^{m-1},\Phi^{m}\right),s.t.{∥\Phi^{m}∥}_{l_{2}}=1.$$

Theoretically, with a step size μ, the optimization problem defined by Equation (7) can be solved using the conventional recursive gradient method [43], where the $i^{\mathrm{th}}$ optimization step can be expressed as

(8) $$\begin{array}{cc} \; & X=\Phi^{m,i-1}+\mu \nabla_{\Phi^{m,i-1}}h\left(y_{k}^{m}|P^{m-1},\Phi^{m,i-1}\right),\\ \; & \Phi^{m,i}=\frac{X}{{∥X∥}_{l_{2}}}. \end{array}$$

According to Equation (3), $h\left(y_{k}^{m}|P^{m-1},\Phi^{m}\right),\Phi^{m}$ at any optimization step can be expressed by

(9) $$\begin{array}{cc} h(y_{k}^{m}|P^{m-1},\Phi^{m}) & =-\int p_{r}\left(y_{k}^{m}|P^{m-1},\Phi^{m}\right)\log\left[p_{r}\left(y_{k}^{m}|P^{m-1},\Phi^{m}\right)\right]dy_{k}^{m}\\ \; & =-\int [\sum_{l=1}^{N_{b}}P_{l}^{m-1}f_{y,l,m}\left(y_{k}^{m}\right)]\log[\sum_{l=1}^{N_{b}}P_{l}^{m-1}f_{y,l,m}\left(y_{k}^{m}\right)]dy_{k}^{m}. \end{array}$$

Using the Taylor expansion of the logarithmic item at $y_{k}^{m}=0$, as $y_{k}^{m}$ is a mixture of Gaussian components with the means of zeros, Equation (9) can be approximated as

(10) $$\begin{array}{cc} h(y_{k}^{m}|P^{m-1},\Phi^{m}) & =-\int [\sum_{l=1}^{N_{b}}P_{l}^{m-1}f_{y,l,m}\left(y_{k}^{m}\right)]\{\log[\sum_{l=1}^{N_{b}}P_{l}^{m-1}f_{y,l,m}\left(0\right)]+\epsilon \left(0\right)y_{k}^{m}+\ldots \}dy_{k}^{m}\\ \; & \approx -\log[\sum_{l=1}^{N_{b}}P_{l}^{m-1}f_{y,l,m}\left(0\right)]\\ \; & =-\log[\sum_{l=1}^{N_{b}}\frac{P_{l}^{m-1}}{\pi \Phi^{m}\left(\Xi_{s,l}+\sigma_{n}^{2}I_{R}\right){\Phi^{m}}^{H}}]. \end{array}$$

where $\epsilon \left(0\right)=\nabla_{y_{k}^{m}}\log\left[\sum_{l=1}^{N_{b}}P_{l}^{m-1}f_{y,l,m}\left(y_{k}^{m}\right)\right]{|}_{y_{k}^{m}=0}$.

Therefore, it can be approximated that

(11) $$\begin{array}{cc} \nabla_{\Phi^{m}}h\left(y_{k}^{m}|P^{m-1},\Phi^{m}\right) & \approx -\nabla_{y_{k}^{m}}\log[\sum_{l=1}^{N_{b}}\frac{P_{l}^{m-1}}{\pi \Phi^{m}\left(\Xi_{s,l}+\sigma_{n}^{2}I_{R}\right){\Phi^{m}}^{H}}]\\ \; & =-\frac{\sum_{l=1}^{N_{b}}P_{l}^{m-1}\nabla_{\Phi^{m}}\left\{{\left[\pi \Phi^{m}\left(\Xi_{s,l}+\sigma_{n}^{2}I_{R}\right){\Phi^{m}}^{H}\right]}^{-1}\right\}}{\sum_{l=1}^{N_{b}}P_{l}^{m-1}{\left[\pi \Phi^{m}\left(\Xi_{s,l}+\sigma_{n}^{2}I_{R}\right){\Phi^{m}}^{H}\right]}^{-1}}\\ \; & =-\frac{\sum_{l=1}^{N_{b}}P_{l}^{m-1}{\left[\Phi^{m}\left(\Xi_{s,l}+\sigma_{n}^{2}I_{R}\right){\Phi^{m}}^{H}\right]}^{-2}\Phi^{m}\left(\Xi_{s,l}+\sigma_{n}^{2}I_{R}\right)}{\sum_{l=1}^{N_{b}}P_{l}^{m-1}{\left[\Phi^{m}\left(\Xi_{s,l}+\sigma_{n}^{2}I_{R}\right){\Phi^{m}}^{H}\right]}^{-1}}. \end{array}$$

### 3.2. Adaptive Measurement Kernel Design Using the DNN

**Figure 2:**
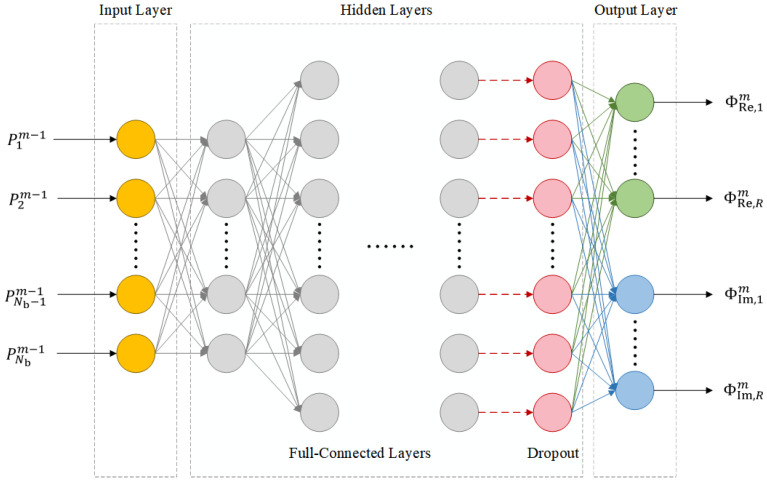
The proposed DNN structure to conduct the adaptive measurement kernel design.

Theoretically, the measurement kernel design can be conducted using Equations (8) and (11). However, the heavy iterative calculations are usually of high complexity and time-consuming. To achieve high efficiency in the non-cooperative analysis of the FHSS signals, the fully connected DNN shown in Figure 2 is proposed specifically in this paper to replace the iterative optimization process.

As shown in Figure 2, the input and output of the proposed DNN are the posterior probabilities of the sub-band occupation and the coefficients in the designed measurement kernel, respectively. As the measurement kernels are complex-valued, the output layer of the DNN has 2R nodes, with *R* nodes (ΦRe,1m,…,ΦRe,Rm in Figure 2) representing the real part of the measurement kernel and *R* nodes (ΦIm,1m,…,ΦIm,Rm in Figure 2) representing the imaginary part. Four hidden layers are included in the DNN, with their widths as 160, 640, 640, and 640, respectively. The final hidden layer is connected to a dropout layer with a dropout rate of 0.75, which is used in both the training and the evaluation stages to enhance the robustness of the generated measurement kernels. The DNN uses ReLU as the activation function in the hidden layers and employs a combination of randomly generated sub-band posterior probabilities and those generated during the conventional gradient descent iterations as the training data. During the training process of the DNN, the negativity of conditional differential entropy $h(y_{k}^{m}|P^{m},\Phi^{m})$ approximated in Equation (10) is used as the penalty function.

## 4. Carrier and DOA Estimations of the FHSS Signal

### 4.1. Estimation of the Carrier

During the adaptive measurement process, the posterior probabilities of sub-band occupation are obtained. In order to localize the carrier precisely, the sub-band with the highest occupation probability is further divided into *W* frequency slots. Subsequently, a further posterior probability calculation of occupation over these *W* slots is conducted for each of the antenna elements, using

(12) $$P_{L,w}^{k}=\frac{P_{L,w,\mathrm{ini}}^{k}f_{y_{k},L,w}\left(y_{k}\right)}{\sum_{w=1}^{W}P_{L,w,\mathrm{ini}}^{k}f_{y_{k},L,w}\left(y_{k}\right)},$$

where *L* represents the sub-band index with the highest posterior probability; $y_{k}={\left[y_{k}^{1},y_{k}^{2},\ldots ,y_{k}^{M}\right]}^{T}$ denotes the measurement data from the $k^{\mathrm{th}}$ antenna element; $P_{L,w}^{k}$ represents the posterior probability that the $w^{\mathrm{th}}$ slot within the $L^{\mathrm{th}}$ sub-band is occupied, given the measurement data $y_{k}$ and the case that the $L^{\mathrm{th}}$ sub-band is occupied; $P_{L,w,\mathrm{ini}}^{k}=\frac{1}{W}$ is the prior probability that the $w^{\mathrm{th}}$ slot is occupied; $f_{y_{k},L,w}\left(y_{k}\right)$ is the PDF of $y_{k}$, given that the signal falls in the $w^{\mathrm{th}}$ slot of the $L^{\mathrm{th}}$ sub-band; and $f_{y_{k},L,w}\left(y_{k}\right)$ is a Gaussian function. By ignoring the correlations among measurements at different steps, the entries of $y_{k}$ are regarded to be independently distributed with zero mean. The variance of the $m^{\mathrm{th}}$ entry in $y_{k}$, i.e., $y_{k}^{m}$, equals $\Phi^{m}\left(\Xi_{s,L,w}+\sigma_{n}^{2}I_{R}\right){\Phi^{m}}^{H}$, where $\Xi_{s,L,w}$ stands for the covariance of a white Gaussian signal within the $w^{\mathrm{th}}$ slot of the $L^{\mathrm{th}}$ sub-band.

For each antenna element, the frequency slot occupation is estimated independently within the $L^{\mathrm{th}}$ sub-band based on the maximum a posterior criterion:

(13) $${\hat{w}}_{k}={\mathrm{argmax}}_{w}P_{L,w}^{k}.$$

Then, the estimated carrier is obtained as the average of the center frequencies in the estimated slots over the antenna elements.

### 4.2. Estimation of the DOA

As discussed above, the envelope variation can be neglected in an instantaneous narrow-band signal, compared to the phase delay introduced by the carrier frequency and the time delay on the antenna array elements. Therefore, the compressed samples at the $m^{\mathrm{th}}$ antenna element can be expressed as

(14) $$y^{m}={\left[y_{1}^{m},y_{2}^{m},\ldots ,y_{K}^{m}\right]}^{T}={\left[\Phi^{m}\left(s^{m}a^{T}\left(\Omega \right)+N^{m}\right)\right]}^{T}=a\left(\Omega \right){\left(\Phi^{m}s^{m}\right)}^{T}+{\left(\Phi^{m}N^{m}\right)}^{T},$$

where $a\left(\Omega \right)={\left[a_{1}\left(\Omega \right),a_{2}\left(\Omega \right),\ldots ,a_{K}\left(\Omega \right)\right]}^{T}$ and $N^{m}=\left[n_{1}^{m},n_{2}^{m},\ldots ,n_{K}^{m}\right]$ represent the steering vector and the collection of the uncompressed noise at the $m^{\mathrm{th}}$ measurement, respectively.

With the independence of the signal and the additive noise at each antenna element, and assuming that the signal is zero mean, the covariance matrix of the measurements at the antenna array can be obtained using the following statistical expectation:

(15) $$\begin{array}{cc} R_{\mathrm{yy}} & =E_{m}\left\{y^{m}{y^{m}}^{H}\right\}\\ \; & =a\left(\Omega \right)E_{m}\left\{{\left(\Phi^{m}s^{m}\right)}^{T}{\left(\Phi^{m}s^{m}\right)}^{*}\right\}a^{H}\left(\Omega \right)+E_{m}\left\{{\left(\Phi^{m}N^{m}\right)}^{T}{\left(\Phi^{m}N^{m}\right)}^{*}\right\}, \end{array}$$

where ${\left(\cdot \right)}^{*}$ stands for the conjugate operation and ${\left(\Phi^{m}s^{m}\right)}^{T}{\left(\Phi^{m}s^{m}\right)}^{*}$ is a scalar. Therefore, $E_{m}\left\{{\left(\Phi^{m}s^{m}\right)}^{T}{\left(\Phi^{m}s^{m}\right)}^{*}\right\}$ can be represented by a positive constant η. In addition, as $\Phi^{m}$ (m=1,2,…,M) is a normalized vector and the noises received by different antenna elements are independent, the entries in the vector ${\left(\Phi^{m}N^{m}\right)}^{T}$ are identically and independently Gaussian-distributed with zero means and the variances of $\sigma_{n}^{2}$. Therefore, Equation (15) can be further derived as

(16) $$R_{\mathrm{yy}}=\eta a\left(\Omega \right)a^{H}\left(\Omega \right)+\sigma_{n}^{2}I_{K},$$

where $I_{K}$ is the *K* × *K* identity matrix.

From Equation (16), it can be further observed that the theoretical eigenvalues of $R_{\mathrm{yy}}$ include a value of $\eta +\sigma_{n}^{2}$ and K−1 repeated values of $\sigma_{n}^{2}$. Consequently, by collecting the compressed measurements at each antenna element, an estimate of the covariance matrix $R_{\mathrm{yy}}$ can be obtained. Then, by selecting the smallest K−1 eigenvalues and corresponding eigenvectors to form the noise subspace $\hat{G}$, the DOA of the source can be estimated by finding the peak of the MUSIC spectrum function:

(17) $$p\left(\Omega \right)=\frac{1}{a^{H}\left(\Omega \right)\hat{G}{\hat{G}}^{H}a\left(\Omega \right)}.$$

With the adaptive measurement kernel design, the measurement kernels can be increasingly coherent with the signal. Therefore, ${\left(\Phi^{m}s^{m}\right)}^{T}{\left(\Phi^{m}s^{m}\right)}^{*}$ is able to converge to a relatively high value. This ensures the separation of the eigenvalues $\eta +\sigma_{n}^{2}$ and $\sigma_{n}^{2}$. Consequently, the noise subspace estimation performance of $R_{\mathrm{yy}}$ and the DOA estimation performance are also ensured.

## 5. Simulations

In order to verify the performance of the proposed method, simulations were conducted using Gaussian-filtered binary frequency-shift keying FHSS signals specified by the Bluetooth standard [44]. The signal during each hopping cycle can be expressed as follows:

(18) $$s\left(t\right)=\sqrt{\frac{E_{s}}{T_{s}}}\exp\{j2\pi [f_{c}t+\varsigma \sum_{r=1}^{N_{s}}b_{r}g\left(t-rT_{s}\right)]\},$$

where $T_{s}$ and $E_{s}$ represent the symbol period and the signal energy in the symbol period, respectively; ς denotes the modulation index of the FHSS signal and $N_{s}$ donates the number of symbol periods within a hopping cycle; $b_{r}$ is the $r^{\mathrm{th}}$ (1 ≤ *r* $\leq N_{s}$) symbol content in a hopping cycle, which takes either of the two values: −1 and 1; and g(t) is the phase pulse function and can be expressed as

(19) $$g\left(t\right)=\frac{Q\left[\alpha (t-\frac{T_{s}}{2})\right]-Q\left[\alpha (t+\frac{T_{s}}{2})\right]}{2T_{s}},$$

where $\alpha =\frac{\pi }{T_{s}\sqrt{\log(2)}}$ and $Q\left(x\right)=\int_{x}^{\infty }\frac{\exp(-\frac{x^{2}}{2})}{\sqrt{2\pi }}$.

As specified by the Bluetooth standard, the frequency-hopping range of the signal was between 2.402 GHz and 2.480 GHz, including 79 channels with a bandwidth of 1 MHz for each channel. To analyze, the simulated non-compressed sampling rate was $F_{nyq}=80$ MHz, based on the band-pass Nyquist sampling theory. The frequency-hopping cycle and the symbol period were set to 625 μs and 1 μs, respectively. To conduct compressed sensing with the proposed method, an observation period of 80 μs was used. In practical implementations, the compression ratio and the observation period can be adjusted to provide enough measurements within a frequency-hopping cycle, even for fast frequency-hopping signals. For simplification, we further assumed that no frequency hop occurred during an observation period.

To implement the proposed method, a uniform linear antenna array (ULA) with 10 elements was used. The distance between the adjacent antenna elements was $\frac{0.5c}{2.5\mathrm{GHZ}}=60\;\mathrm{mm}$, with *c* representing light speed. The DOA of the FHSS signal used in each simulation was randomly selected, with a uniform distribution from −90 to 90 degrees. The entire range from 2.4015 GHz to 2.4805 GHz was divided into 20 sub-bands (i.e., $N_{b}$ = 20).

The training of the DNN to perform the adaptive measurement kernel design was conducted using the Python package of Pytorch 1.10.2 in the GPU version [45], which was installed on a workstation with the GeForce RTX 4060 GPU and 32 GB RAM. A total of 40,000 samples, which included randomly generated sub-band posterior probabilities and sub-band posterior probabilities randomly selected during the gradient descent iteration process, were taken as the training data. The ratio of the randomly generated samples to the selected samples from iterative simulations was 1:3. The adaptive moment estimation was used as the optimizer with a step size of $10^{-5}$, and a total number of 500 epochs were conducted in the network training process.

At the carrier-estimation step, a sub-band was further divided into four slots (i.e., W=4). In addition, the compression ratio was set to R=10, resulting in 640 compressed measurements for each antenna element during an entire observation period. The SNR, defined as $SNR=\frac{E_{s}}{\sigma_{n}^{2}T_{s}}$, was taken between −25 dB and −5 dB.

**Figure 3:**
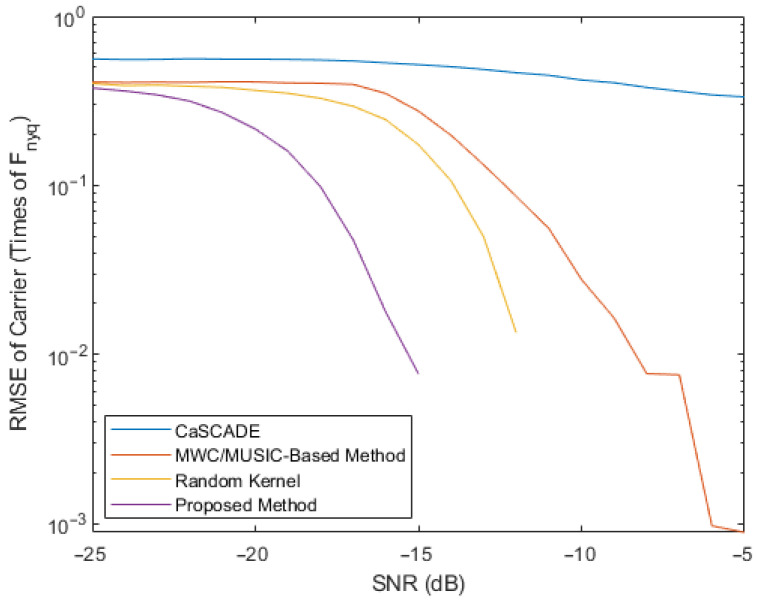
RMSE comparison of the estimated carriers.

**Figure 4:**
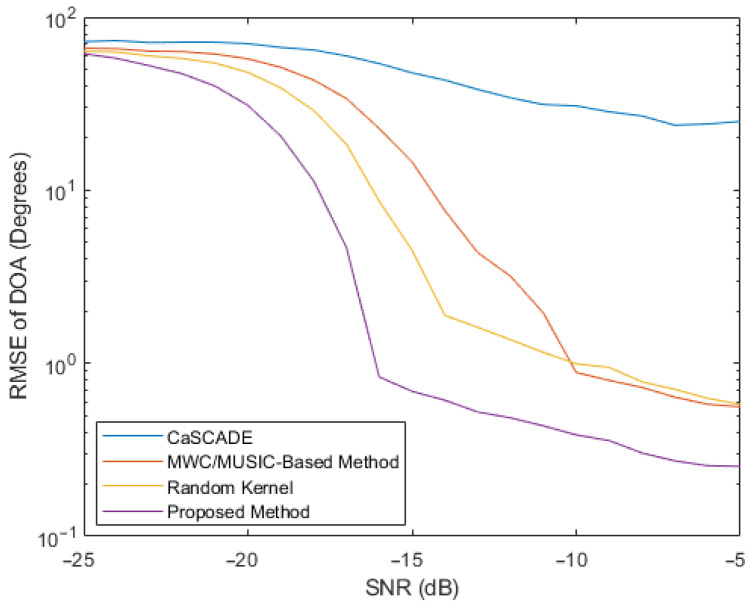
RMSE comparison of the estimated DOAs.

The resulting root mean square error (RMSE) of the estimated carrier versus the SNR and the RMSE of the estimated DOA versus the SNR are plotted in Figure 3 and Figure 4, respectively. To generate each point in the plots, 10,000 simulations were performed. In addition, the CaSCADE algorithm proposed in [33] and the MWC/MUSIC-based method proposed in [35] were also simulated, with the corresponding results shown in Figure 3 and Figure 4, where the same observation periods and the same numbers of compressed measurements were used. For the MWC/MUSIC-based method, the same ULA for the proposed method was implemented. For the CaSCADE algorithm, an L-shaped antenna array that implemented the modified MWC was required. Therefore, an L-shaped antenna array with six antenna elements in the vertical and horizontal directions, including a common antenna element at the origin, was used. Each antenna element was connected to one channel of the MWC. The distance between adjacent antenna elements of the L-shaped antenna array was the same as that used in the ULA. Moreover, in order to validate the advantage of the adaptively designed measurement kernels, the proposed framework with normalized random measurement kernels was also simulated, with the results shown in Figure 3 and Figure 4.

From Figure 3 and Figure 4, it can be observed that the estimated RMSEs of the frequency and the DOA for each method in comparison decreased with the increase of SNR. Compared to the MWC/MUSIC-based method with the same number of antenna elements and the CaSCADE algorithm with even one additional antenna element, the proposed framework with random measurement kernels obtained improved RMSEs of frequency estimations at median and high SNR values and improved RMSEs of DOA estimations at median SNR values. However, at relatively low SNR values, it did not provide significantly improved performance for the estimations of both parameters, compared to the MWC/MUSIC-based method. In contrast, at both low and high SNR values, the proposed adaptive compressed measurement and signal analysis method achieved the lowest RMSE values for both the frequency and DOA estimations of the FHSS signals, compared to those two methods in the literature. In particular, to achieve the same carrier and DOA estimation accuracy, the proposed adaptive method can even work at an SNR value more than 5 dB lower, compared to the MWC/MUSIC-based method.

**Figure 5:**
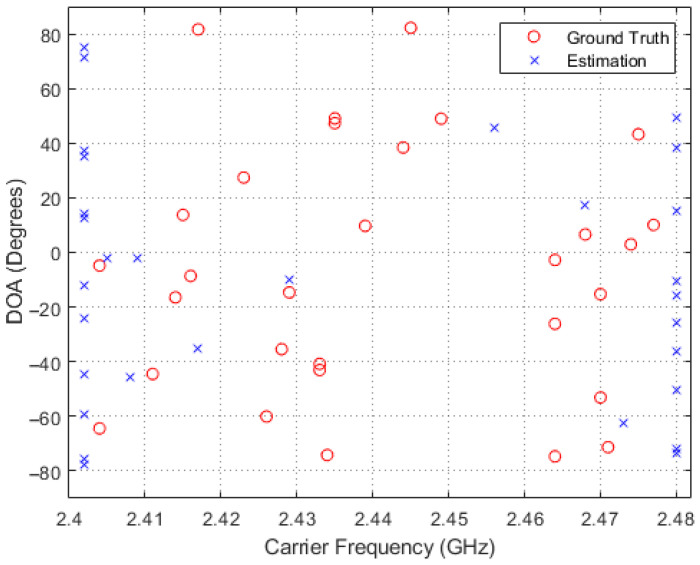
Sampled comparisons between the carriers and DOAs in ground truth and those estimated using the CaSCADE algorithm.

**Figure 6:**
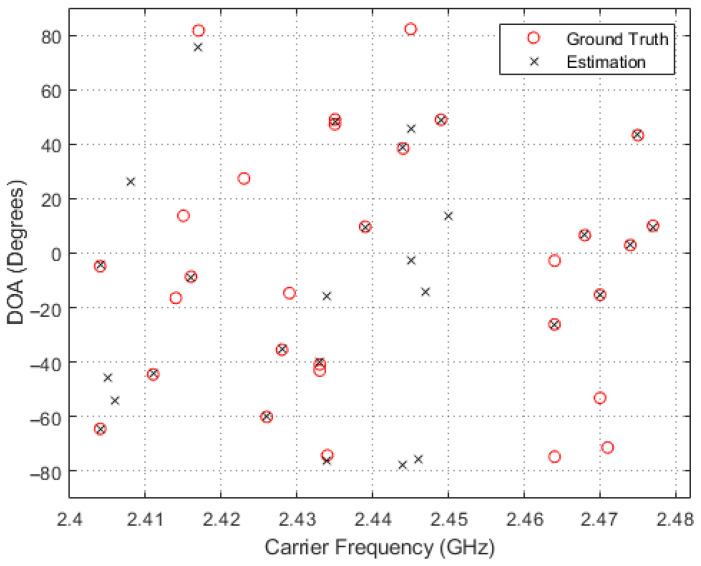
Sampled comparisons between the carriers and DOAs in ground truth and those estimated using the MWC/MUSIC-based method.

**Figure 7:**
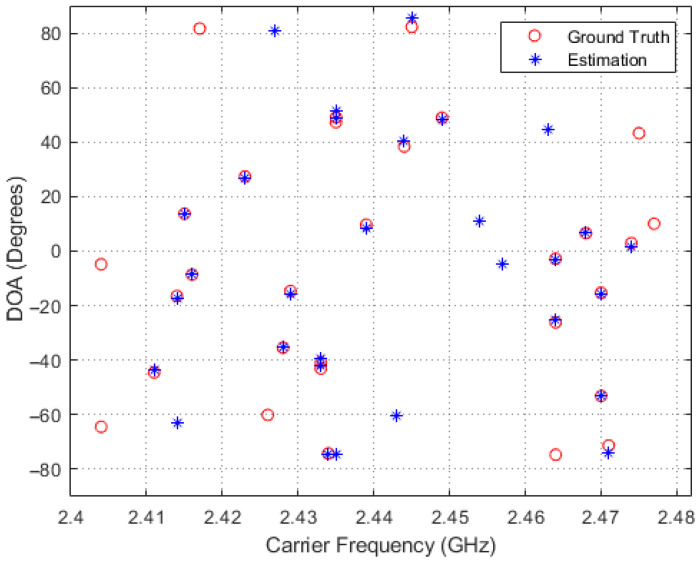
Sampled comparisons between the carriers and DOAs in ground truth and those estimated using the proposed framework with random measurement kernels.

**Figure 8:**
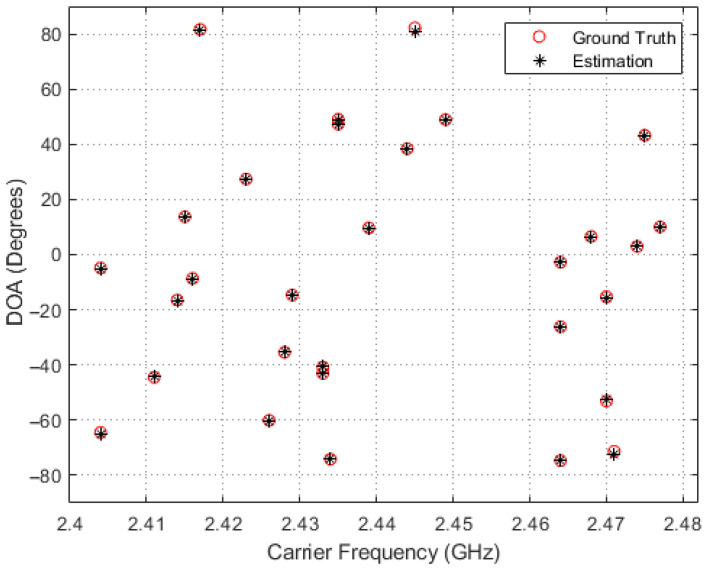
Sampled comparisons between the carriers and DOAs in ground truth and those estimated using the proposed adaptive method.

In order to obtain a deeper insight into the simulation results, scatter plots of the estimated and true carrier and DOA values for the four methods in comparison are shown in Figure 5, Figure 6, Figure 7 and Figure 8, where 30 simulation results for each of the four methods with randomly generated frequencies and DOAs at SNR = −15 dB were included. From those four figures, it can be seen that the estimated frequencies and DOAs using the proposed adaptive method were the closest to the ground truth.

Furthermore, to verify the efficiency of the proposed method, a timing comparative study between the deep learning-based measurement kernel design method and the conventional iterative measurement kernel optimization specified in Equation (8) was conducted. In this study, the experimental parameters remained and an SNR of −18 dB was used. To achieve the performance of the deep learning-based method, 20,000 optimization steps are needed, according to the simulation trials. The statistical comparison of time cost for the two methods over 500 processing cycles, including the adaptive measurement kernel designs, the sub-band occupation probability updates, and the carrier/DOA estimation in each cycle, is shown in Table 1. In this comparison, the maximum, minimum, and mean values of the time costs are shown.

From Table 1, it can be seen that the time cost of the proposed deep learning-based method can be 0.33% of the conventional iterative optimization method for adaptive measurements of FHSS signals. In particular, the shortest time cost of the deep learning-based method was only 8.9006 s, which is hundreds of times’ acceleration, compared to the conventional iterative optimizations. Therefore, it is verified that the proposed deep learning-based method shows improved efficiency in adaptive measurements.

## 6. Conclusions

In this paper, a non-cooperative method to estimate the carrier frequency and the DOA of FHSS signals was proposed. By this method, the signal measurements can be conducted with antenna arrays at much-compressed rates compared to those determined by the Nyquist sampling theory and entire FHSS ranges. Unlike conventional compressed sensing, the measurements were done adaptively, with the measurement kernels designed based on the TSI optimization and the analysis of the measurement data that had been obtained. In order to ensure the efficiency of the measurement kernel designing process, a DNN was trained so that the repeated online recursive optimizations of the measurement kernels were replaced by one-time offline training and repeated direct implementation of the DNN.

Through simulations, frequency and DOA estimation accuracy were also validated by comparison with the methods in the literature and the proposed compressed framework with conventional random measurement kernels. In addition, the efficiency of the proposed method was verified.

## Figures and Tables

**Table 1:** Statistical comparison of time cost between the proposed adaptive method and the conventional iterative method.

	Mimimum Time Cost per Estimation (s)	Averaged Time Cost per Estimation (s)	Maximum Time Cost per Estimation (s)
**The Proposed Deep Learning-Based Method **	8.9006	9.8371	14.2708
**The Conventional Iterative Optimization Method**	2850.7	2968.4	3374.0

## Data Availability

The original contributions presented in the study are included in the article material; further inquiries can be directed to the corresponding author.

## Abbreviations

The following abbreviations are used in this manuscript:

SS	spread spectrum
FHSS	frequency-hopping spread spectrum
TSI	task-specific information
CS	compressed sensing
MWC	modulated wideband converter
MUSIC	multiple signal classification
SNR	signal-to-noise ratio
GPU	graphics processing unit
DNN	deep neural network
PDF	probability density function
ULA	uniform linear antenna array
RMSE	root mean squared error
